# Neurostimulation in Addictions: a retrospective study in patients with Cocaine Use Disorder undergoing a rTMS protocol

**DOI:** 10.1192/j.eurpsy.2025.202

**Published:** 2025-08-26

**Authors:** A. Gual, P. Lusilla

**Affiliations:** 1 Health and Addictions Research Group, Fundació Clínic - Idibaps; 2 Centre Bonanova; 3 Psychiatry Department, Hospital Vall d’Hebron, Barcelona, Spain

## Abstract

**Abstract:**

There are clear unmet medical needs in the treatment of Cocaine Use Disorder (CUD), since there are no pharmacological treatments approved. The neurobiological circuitry of addiction has been described in recent years, and it provides a solid rationale to target specific brain regions to treat addictive behaviors, including CUD. The stimulation of the left Dorsolateral Prefrontal Cortex (DLPFC) with repetitive transcranial magnetic stimulation (rTMS) has proved to reduce craving for various drugs, including cocaine.

We present the results of a retrospective study performed in a private setting with 93 patients with CUD, who were treated with rTMS following the protocol from Terraneo et al. In 12 weeks patients received 32 sessions of 2400 pulses (100% MT; 10 Hz; 60 pulses per train, 15 seconds interval; 40 trains per session), for a total of 76800 pulses.

The main outcome was total abstinence of cocaine (self-reported plus urinalysis). Abstinence rates at days 30, 60 and 90 were 55,9%; 40,8%; and 34,4% respectively.

Psychiatric comorbidities (insomnia, depression and anxiety) were also assessed with validated questionnaires. Drop-out was directly related to recent cocaine use, but initial levels of psychological distress did not predict drop-out.

No relevant side effects were reported. Mild and transient headache was reported by a few patients after the first session.

In conclusion rTMS was well tolerated, drop out rates were high and 34% of the patients remained abstinent after the 90 days treatment period.

**Disclosure of Interest:**

None Declared

